# Ramadan is not associated with increased infection risk in Pakistani and Bangladeshi populations: Findings from controlled interrupted time series analysis of UK primary care data

**DOI:** 10.1371/journal.pone.0262530

**Published:** 2022-01-13

**Authors:** Munerah Almulhem, Rasiah Thayakaran, Shahjehan Hanif, Tiffany Gooden, Neil Thomas, Jonathan Hazlehurst, Abd A. Tahrani, Wasim Hanif, Krishnarajah Nirantharakumar

**Affiliations:** 1 Institute of Applied Health Research, College of Medical and Dental Sciences, University of Birmingham, Birmingham, United Kingdom; 2 Life and Medical Sciences, University College London, London, United Kingdom; 3 Institute of Metabolism and Systems Research, University of Birmingham, Birmingham, United Kingdom; 4 Centre for Endocrinology, Diabetes and Metabolism, Birmingham Health Partners, Birmingham, United Kingdom; 5 Diabetes Department, University Hospitals Birmingham NHS Foundation Trust, Birmingham, United Kingdom; Shahjalal University of Science and Technology (SUST), BANGLADESH

## Abstract

**Background:**

The effect of fasting on immunity is unclear. Prolonged fasting is thought to increase the risk of infection due to dehydration. This study describes antibiotic prescribing patterns before, during, and after Ramadan in a primary care setting within the Pakistani and Bangladeshi populations in the UK, most of whom are Muslims, compared to those who do not observe Ramadan.

**Method:**

Retrospective controlled interrupted time series analysis of electronic health record data from primary care practices. The study consists of two groups: Pakistanis/Bangladeshis and white populations. For each group, we constructed a series of aggregated, daily prescription data from 2007 to 2017 for the 30 days preceding, during, and after Ramadan, respectively.

**Findings:**

Controlling for the rate in the white population, there was no evidence of increased antibiotic prescription in the Pakistani/Bangladeshi population during Ramadan, as compared to before Ramadan (IRR: 0.994; 95% CI: 0.988–1.001, p = 0.082) or after Ramadan (IRR: 1.006; 95% CI: 0.999–1.013, p = 0.082).

**Interpretation:**

In this large, population-based study, we did not find any evidence to suggest that fasting was associated with an increased susceptibility to infection.

## Introduction

The Muslim population represents nearly 1.6 billion people, comprising 23% of the global population in 2010 [1]. As part of the five pillars of Islam, it is conventional and commonplace for adult Muslims to fast for the holy month of Ramadan. Ramadan involves abstaining from the consumption of food, drink, and oral medications between sunrise and sunset for the entire month. Ramadan is the ninth month of the lunar calendar and depending on the time of year this occurs, as well as the geographical location, practising Muslims may go exceptionally long hours without basic, yet vital, necessities for optimal health. While the impact of Ramadan fasting on metabolic parameters and conditions have been moderately investigated [2], other health outcomes during this annual event are poorly understood.

The impact of Ramadan fasting specifically on infection is unclear. However, it is imperative to ascertain the risk of infection in order to provide effective healthcare and advice to this potentially vulnerable population. This is particularly essential during the COVID-19 pandemic, where minority ethnic groups are at an increased risk for adverse medical complications [3]. Though limited, there is evidence that Ramadan fasting transiently improves immune response [4]. For instance, some studies report reduced levels of leukocytes and circulating proinflammatory cytokines (IL-1β, IL-6, and TNF-α) [5, 6]. However, prolonged fasting can lead to dehydration which may increase the risk of infection, particularly in warm weather [7]. In addition to altering their daily routine by only eating two meals a day (‘suhur’ before dawn and ‘iftar’ after dusk), practising Muslims also attend additional religious gatherings and social parties during Ramadan, thereby sleeping less at night [8–10]. Insufficient sleep has been associated with reduced immune responses [11], and extensive physical interaction with others can increase the risk of exposure to an infectious agent. The many social and religious gatherings taking place during Ramadan constitute an additional risk factor for infection [3].

Type 2 diabetes, another risk factor for developing severe illness from COVID-19, is highly prevalent in the Muslim population [12, 13]. Extended fasting can result in poor glycaemic control in diabetics, which has been shown to reduce T cell response, neutrophil function, and humoral immunity, exacerbating the risk of infection during dehydration [14, 15]. Even though Muslims with diabetes are often exempt from taking part in Ramadan fasting, more than half choose to participate in this holy event [16, 17].

The existing systematic review examining the relationship between Ramadan fasting and infectious diseases does not look at the risk of acquiring infection [18]. Instead, the impact of Ramadan fasting on Muslims with chronic infections, such as HIV, was assessed. While this review provides useful recommendations for physicians already treating patients with infectious diseases who are fasting during Ramadan, there are few guidelines to help address the concerns of healthy patients wishing to participate. The guidelines that do exist are predominately based on expert opinion [19]. Given that the Muslim population has a high burden of chronic disease, especially diabetes and cardiovascular disease, and is therefore already at an increased risk of complications and infections, it is critical for healthcare professionals to be able to provide reliable advice regarding fasting during Ramadan [20]. Much uncertainty still exists about the risk of infection during Ramadan. No previous study has been conducted to explore the risk of infection during Ramadan in a predominant Muslim population.

Most of the available evidence is based on small studies or observational studies that lacked a robust design. Conducting a randomized control study is challenging and unethical as Ramadan fasting is a holy practice for Muslims. Meanwhile, recruiting those who are not fasting as a control group in an observational study might bias the findings, as these individuals are likely to be exempted from fasting due to health issues. ITS is an increasingly used design in public health research that adopts an approach that allows for comparisons to be made over time within single populations [21, 22]. Interrupted time series is one of the strongest, quasi-experimental designs to evaluate longitudinal effects exposure, particularly when randomised controlled trials are not possible. However, this design cannot exclude the effect of confounders or events that occurred in the same time period as the exposure under investigation (the so-called history bias), which threaten the internal validity of the study. The literature suggests that adding a control series will minimize this bias and strengthen the study design (controlled interrupted time series) [23].

According to the 2011 UK census, 4.8% of the population identify as Muslim [24]. This number is projected to be as high as 11.3% by 2050 [1]. In the UK, Muslims may fast for nearly 17 hours a day if Ramadan falls during the summer months, which could prove dangerous if temperatures are high. To address the lack of information on the effects of prolonged fasting on infection, we aim to describe antibiotic prescribing patterns before, during, and after Ramadan in the Pakistani and Bangladeshi populations (largely Muslim) in the UK, and compare these patterns to the white population (largely non-Muslim).

## Methods

### Study design and data sources

We performed a retrospective controlled interrupted time series analysis using the IQVIA Medical Research Data (IMRD), also referred to as The Health Improvement Network (THIN) database. This is an electronic primary care patient record in the UK. THIN is a large UK general practice database that contains anonymised longitudinal patient records from over 750 general practices (about 6% of the population at a given time). THIN provides longitudinal records with data on socio-demographic characteristics, diagnoses, medical test results, prescriptions, and additional information (e.g., lifestyle). Diagnoses are recorded as Read Codes, a hierarchal coding system. Prescriptions are entered using Multilex codes issued by First Databank; these can be easily linked to British National Formulary (BNF) codes. THIN data access was provided by IQVIA to the University of Birmingham. The study protocol was reviewed and approved by an independent scientific review committee (reference number: 19THIN044). IQVIA Medical Research Data incorporates THIN data, a Cegedim Database. References made to THIN are intended to be descriptive of the data asset licensed by IQVIA. Our study uses anonymised data provided by patients as a part of their routine primary care. The Scientific Review Committee (IQVIA) approved the study protocol (no. SRC19THIN044) before its start. Informed consent was not required in this study as the data were anonymized from the provider.

To ensure the quality of the recorded data, we included only general practices that were using electronic medical record software for at least one year, and that also had acceptable mortality recordings for at least one year [25].

### Study period

The study includes the time periods before, during, and after Ramadan each year from 2007 to 2017. The timing of Ramadan is based on the lunar calendar and calendar months can last for 29 or 30 days, depending on the moon’s phases. For this study, we standardised the months to 30 days. We marked the start of Ramadan in each year and based on that date, identified 30 days before, 30 days during, and 30 days after. We only included 90 days for each year of the study.

### Population

**Fig 1:**
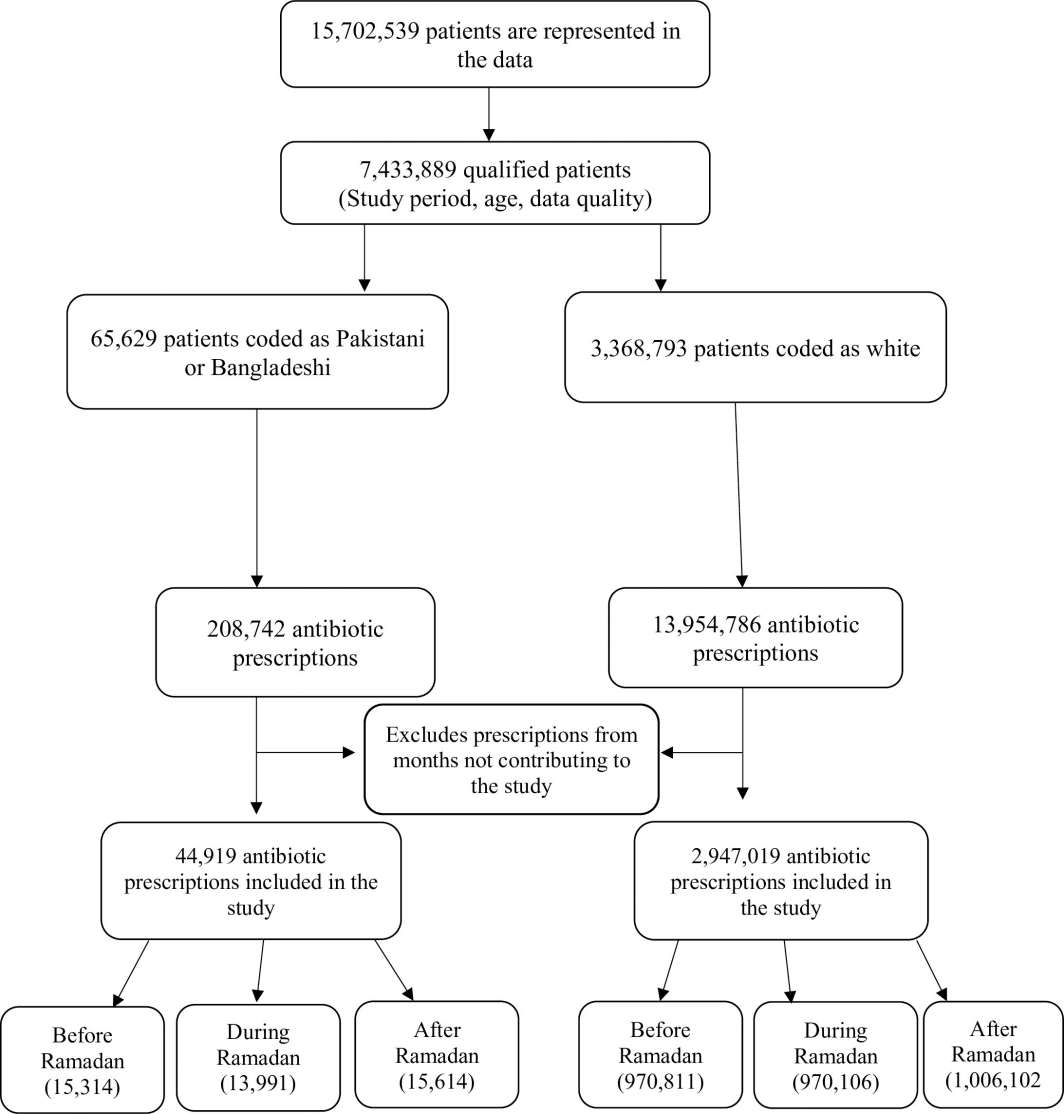
Flow diagram of the study data.

The study consists of two groups. The first is the targeted group: Pakistanis and Bangladeshis representing the Muslim population in this study. Pakistanis and Bangladeshis represent the largest Muslim ethnic groups in the UK, constituting 38% and 15%, respectively, of the overall Muslim population [26]. These populations have a high burden of chronic disease, in particular type 2 diabetes (T2D) and cardiovascular disease; they also have an increased risk of infection and disease-specific complications [20]. The second group is the white population representing the control group, which predominantly does not observe Ramadan. These cohorts were identified through relevant ethnicity codes (Fig 1). The patients entered the cohort one year after registration with the practice or after the date that a practice became eligible to contribute, whichever was the most recent; this ensured data quality and sufficient covariate recording.

### Study variables

We measured antibiotic prescriptions before, during, and after Ramadan. All antibiotic prescriptions recorded in the databases during this period of interest were retrieved. Baseline data on demographic characteristics were collected at cohort entry.

### Analysis

We used descriptive statistics to identify the basic features of the data from before, during, and after Ramadan. We analysed patterns of antibiotic prescription using interrupted time series (ITS) analysis. ITS analysis is a quasi-experimental design that can evaluate an intervention effect using longitudinal data. The term ‘quasi-experimental’ refers to an absence of randomisation; ITS analysis is a tool for analysing observational data when randomisation or a cohort design are not possible. ITS analysis requires data on continuous or counted outcome measures, summarised at regularly spaced time intervals. This model often uses before-and-after comparisons for underlying trends, and is particularly useful for assessing the impacts of policies or healthcare initiatives. It can provide information on any changes that could have occurred due to the intervention/exposure, whether immediately or over time. Single interrupted time series (SITS) analysis elucidates changes within a group before and after the exposure. Controlled interrupted time series (CITS) analysis, which includes adding a control group, helps separate the effect of the exposure from other confounding effects that could have occurred at the same time [23, 27].

For each group (Pakistani/Bangladeshi and white), we developed a series of 90-day data (30 days before, 30 days during, and 30 days after Ramadan) comprised of aggregated, daily data from 2007 to 2017. We performed two analyses: ITS and CITS. We then explored effect modification by refitting CITS model using the following factors: sex, age (< 60, >60), and diabetes status.

1 Interrupted time series (ITS) analysis using poison regression model.

A minimum of three variables are required for an ITS analysis [28]:

- *T⁠* is the time since the start of the study (e.g., day, month, or year);
- *X*_*t*_ is a dummy (indicator) variable representing the intervention (pre-intervention periods equal 0, otherwise 1); and
- *Y*_*t*_ is the aggregated outcome variable measured at each equally spaced time point *t*.

The following segmented regression model is used for standard ITS analysis [28]:

$$Y_{t}=\beta_{0}+\beta_{1}T+\beta_{2}X_{t}+\beta_{3}TX_{t}+\varepsilon_{t}$$

where *β*_*0*_ represents the baseline level at *T* = 0; *β*_*1*_ represents the change in outcome associated with a time unit increase (representing the underlying pre-intervention trend); *β*_*2*_ is the level of change following the intervention; *β*_*3*_ indicates the change in slope pre- and post-intervention; *ɛ*_*t*_ is an error term.

2 Controlled interrupted time series (CITS) analysis using negative binominal regression model.

A minimum of four variables are required for a controlled interrupted time series (CITS) analysis [28]:

- *T⁠* is the time since the start of the study (e.g., day, month, or year);
- *X*_*t*_ is a dummy (indicator) variable representing the intervention (pre-intervention periods equal 0, otherwise 1);
- *Z*_t_ is a dummy variable to denote the cohort assignment (treatment or control); and
- *Y*_*t*_ is the aggregated outcome variable measured at each equally spaced time point *t*.

The controlled interrupted time series model is indicated by the following equation [28]:

$$Y_{t}=\beta_{0}+\beta_{1}T+\beta_{2}X_{t}+\beta_{3}TX_{t}+\beta_{4}Z_{t}+\beta_{5}Z_{t}T+\beta_{6}Z_{t}X_{t}+\beta_{7}Z_{t}TX_{t}+\varepsilon_{t}$$

where *β*_*0*_ to *β*_*3*_ represents the control group, and *β*_*4*_ to *β*_*7*_ represents the treatment group. *Β*_*4*_ represents the difference in level between treatment and control prior to the intervention. *Β*_*5*_ represents the difference in the slope between treatment and control prior to the intervention. *Β*_*6*_ represents the difference in level between treatment and control in the period immediately following the intervention initiation. *Β*_*7*_ represents the difference between the treatment and control in the slope after the intervention was initiated, compared to the pre-intervention period; *ɛ*_*t*_ is an error term.

Dataset structure for both models is available as supplementary material (S2 Appendix)

The validity of ITS design rest of assumptions that imply that a linear extrapolation of the pre period trendline into the post period provides an unbiased representation of the counterfactual for a treated sample [29], We checked these assumption in addition to seasonality (S1 Appendix).

All parameter estimation for ITS and CITS models are available as (S3 Appendix). We used R for analysis.

## Results

### Study population

Included in the study were 1,097,429 patients and 3,308,463 antibiotic prescriptions. A total of 65,629 patients were coded as Pakistani/Bangladeshi and 3,368,793 patients were coded as white. A total of 2,991,938 antibiotic prescriptions contributed to the study, including 44,919 for the Pakistani/Bangladeshi group and 2,947,019 for the white control group. A total of 18,632 patients contributed to the study, 18,632 Pakistani/Bangladeshi and 1,078,797 White (Table 1). Across the study period the proportion of female prescribed antibiotics is higher in both groups. The mean age at prescription was less in the Pakistani/Bangladeshi population compared to the white population. Compared to the white group higher proportion of patients with diabetes were prescribed antibiotics. Table 2 provides an overview of study populations before, during and after Ramadan.

**Table 1:** Characteristics of aggregated antibiotic prescriptions data for 90 days each year from 2007 to 2017.

	White n = 2,947,019	Pakistani/Bangladeshi n = 44,919
Number of patients contributing to the prescriptions	1,078,797	18,632
Sex		
Male	404,889 (37.5%)	7,838 (42.1%)
Female	673,908 (62.5%)	10,794 (57.9%)
Age*, mean (SD) (all)	56.2 (19.7)	43.99 (16.5)
Male	57.58 (18.8)	46.22 (17.6)
Female	55.52 (20.1)	42.61 (15.6)
Townsend deprivation index score, n (%)		
1(least deprived)	198,963 (18.4%)	1,255 (6.7%)
2	190,832 (17.7%)	1,644 (8.8%)
3	194,640 (18%)	2,600 (14%)
4	176,188 (16.3%)	4,198 (22.5%)
5(Most deprived)	133,305 (12.4%)	4,482 (24.1%)
6 (Missing)	184,869 (17.2%)	4,453 (23.9%)
**Patients with diabetes**	170,428 (15.8%)	5,311 (28.5%)

*Age at prescription.

**Table 2:** Characteristics of aggregated antibiotic prescriptions data from before, during, and after Ramadan each year from 2007 to 2017.

	White			Pakistani/Bangladeshi		
Study period	Before Ramadan n = 970,811	During Ramadan n = 970,106	After Ramadan n = 1,006,102	Before Ramadan n = 15,314	During Ramadan n = 13,991	After Ramadan n = 15,614
Number of patients contributing to the prescriptions	554,780	554,340	569,566	9,666	8,872	9,666
Sex						
Male	199,359 (35.9%)	198,547 (35.8%)	203,587 (35.7%)	3,829 (39.6%)	3,600 (40.6%)	3,878 (40.1%)
Female	355,421 (64.1%)	355,793 (64.2%)	365,979 (64.3%)	5,837 (60.4%)	5,272 (59.4%)	5,788 (59.9%)
Age*, mean (SD) (all)	56.38 (19.6)	56.20 (19.7)	56.09 (19.7)	44.05 (16.4)	43.68 (16.6)	44.21 (16.5)
Male	57.73 (18.7)	57.50 (18.8)	57.50 (18.8)	46.39 (17.6)	45.82 (17.6)	46.42 (17.6)
Female	55.67 (20.)	55.53 (20.1)	55.36 (20.1)	42.64 (15.5)	42.33 (15.8)	42.83 (15.5)
Townsend deprivation index score, n (%)						
1(least deprived)	100,918 (18.2%)	100,780 (18.2%)	102,503 (18%)	665 (6.9%)	593 (6.7%)	635 (6.6%)
2	97,584 (17.6%)	96,982 (17.5%)	99,730 (17.5%)	819 (8.5%)	791 (8.9%)	848 (8.8%)
3	99,947 (18%)	99,782 (18%)	102,928 (18.1%)	1,342 (13.9%)	1,235 (13.9%)	1,339 (13.9%)
4	91,659 (16.5%)	92,064 (16.6%)	94,072 (16.5%)	2,179 (22.5%)	2,033 (22.9%)	2,156 (22.3%)
5 (Most deprived)	70,506 (12.7%)	70,600 (12.7%)	73,379 (12.9%)	2,325 (24%)	2,106 (23.7%)	2,325 (24%)
6 (Missing)	94,166 (17%)	94,132 (17%)	96,954 (17%)	2,336 (24.2%)	2,114 (23.9%)	2,363 (24.4%)
**Patients with diabetes**	56,503 (10.18%)	56,317 (10.16%)	57,608 (10.11%)	1,790 (18.51%)	1,609 (18.13%)	1,751 (18.17%)

*Age at prescription.

### Single interrupted time series analysis

**Fig 2:**
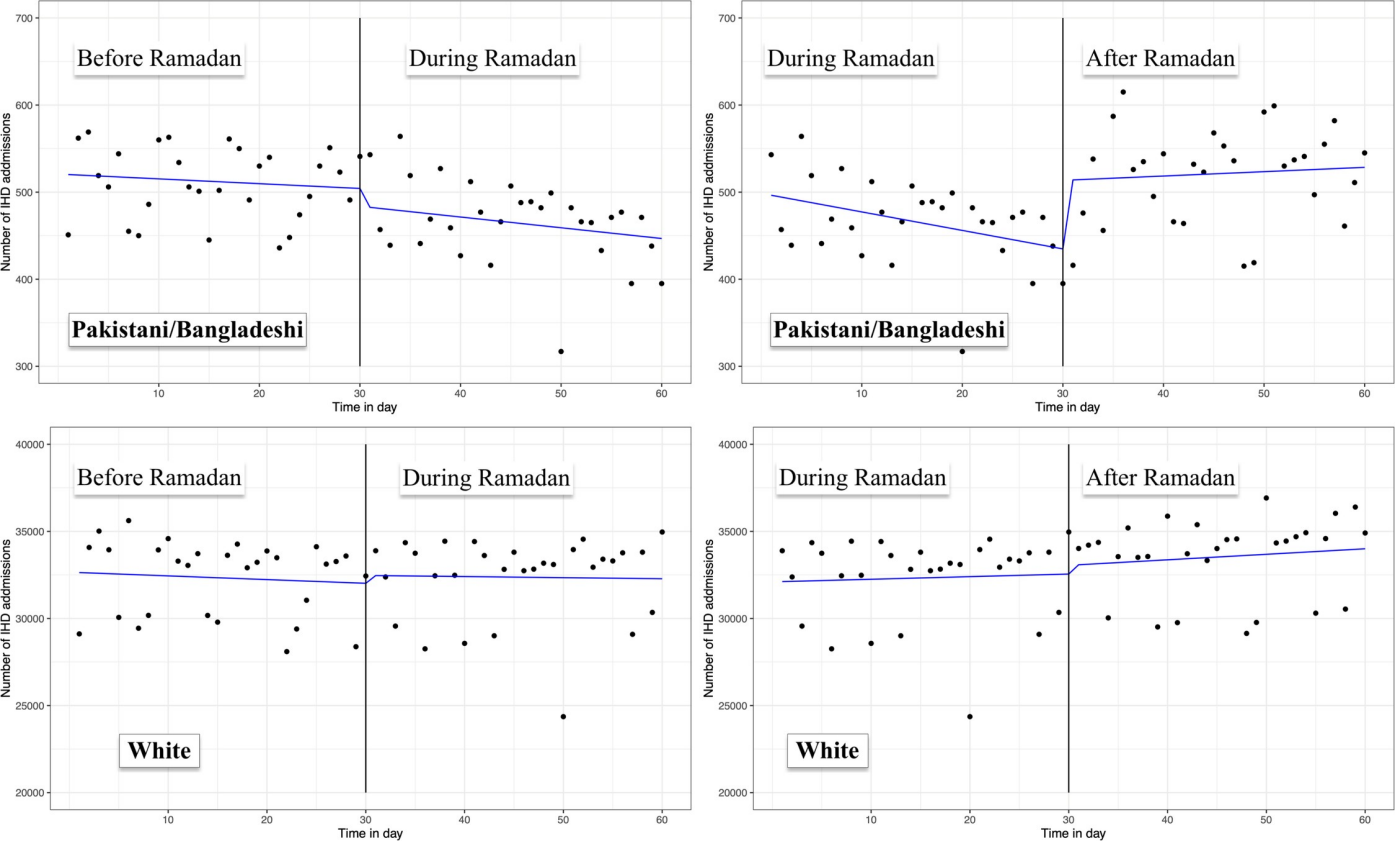
Daily antibiotic prescription patterns (SITS).

Time series analysis did not identify any significant trend change in antibiotic prescriptions during Ramadan (compared to before Ramadan) in the Pakistani/Bangladeshi group (IRR: 0.995; 95% CI: 0.990–1.001, p = 0.089). After Ramadan, there was an increase in antibiotic prescriptions compared to during the Ramadan period (IRR: 1.007,95% CI: 1.001–1.013, p = 0.025). There was no change observed in prescription patterns in the white population during Ramadan (IRR: 1.001; 95% CI%: 0.997–1.006, p = 0.54) or after Ramadan (IRR: 1.001; 95% CI: 0.996–1.005, p = 0.78) (Fig 2, Table 3).

**Table 3:** Findings from the SITS and CITS analyses for the general population.

Parameter	SITS	P-value	Parameter	CITS	P-value
	Incidence Rate Ratio (95% CI)			Incidence Rate Ratio (95% CI)*	
Difference in trend changes before/during Ramadan (Pakistani/Bangladeshi) - ***β*3**	0.995 (95% CI: 0.990–1.001)	0.089	Difference in trend changes between the two groups before/during Ramadan - ***β*7**	0.994 (95% CI: 0.988–1.001)	0.082
Difference in trend changes before/during Ramadan (White)- ***β*3**	1.001 (95% CI: 0.997–1.006)	0.54			
Difference in trend changes during/after Ramadan (Pakistani/Bangladeshi)- ***β*3**	1.007 (95% CI: 1.001–1.013)	0.025	Difference in trend changes between the two during/after Ramadan - ***β*7**	1.006 (95% CI: 0.999, 1.013)	0.082
Difference in trend changes during/after Ramadan (White)- ***β*3**	1.001 (95% CI: 0.996–1.005)	0.78			

*IRR (95%CI) in the Pakistani/Bangladeshi population controlled for the background rate in the white population.

### Controlled interrupted time series analysis

**Fig 3:**
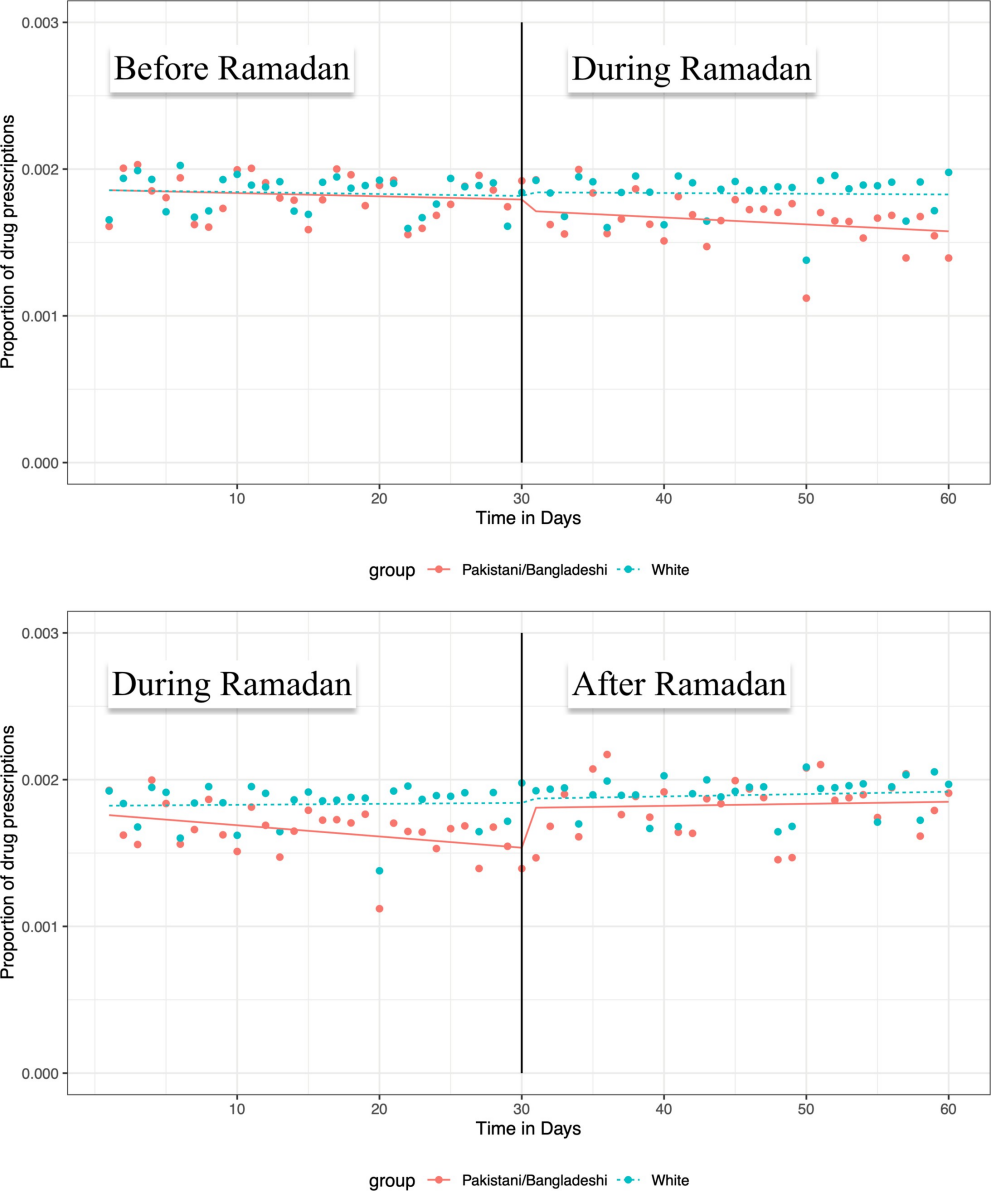
Daily antibiotic prescription patterns in (CITS)*. *Proportion = Number of antibiotic prescriptions/total number of people in that period.

Controlling for the background prescription pattern in the white population did not alter our findings when antibiotic prescriptions during Ramadan in the Pakistani/Bangladeshi group were compared to those before Ramadan (IRR: 0.994; 95% CI: 0.988–1.001, p = 0.082). The significant increase observed in the post-Ramadan period (compared to during the Ramadan period) in the SITS analysis was not evident in the Pakistani/Bangladeshi group when controlled for background prescription rates in the white population (IRR: 1.006; 95% CI: 0.999–1.013, p = 0.082) (Fig 3, Table 3).

### Stratified analysis

**Fig 4:**
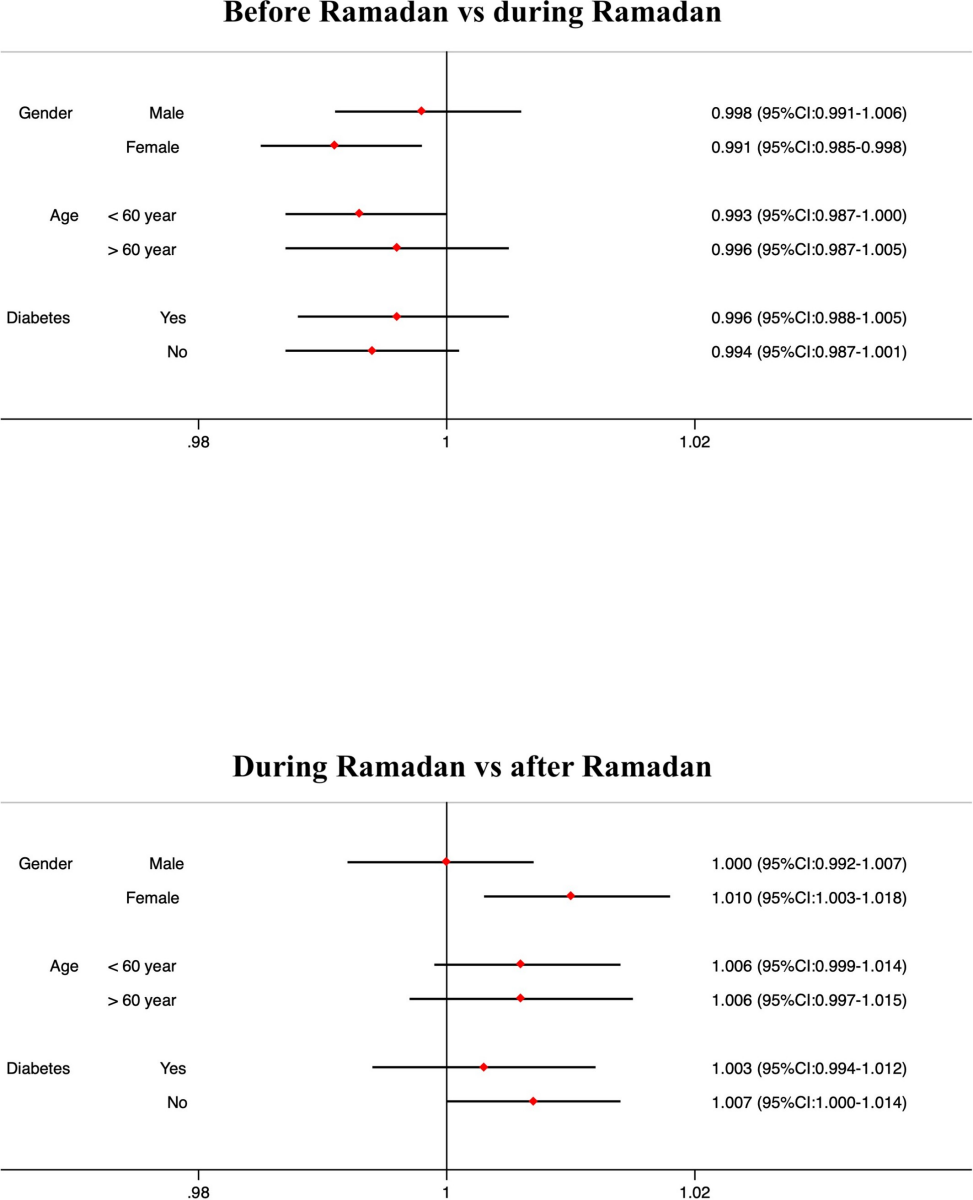
Incidence rate ratios with 95% confidence intervals for the stratified groups in the Pakistani/Bangladeshi population, controlled for the background rate in the white population.

Fig 4 shows the IRR (95% CI) for the stratified groups in the Pakistani/Bangladeshi population, controlled for the background rate in the white population. Females showed a significant decrease in antibiotic prescriptions during Ramadan compared to before (IRR: 0.991; 95% CI: 0.985–0.998). However, females showed an increase in prescriptions after Ramadan compared to during (IRR: 1.010; 95% CI:1.004–0.018). We observed no changes in prescription pattern for men during Ramadan (IRR: 0.998; 95% CI: 0.991–1.006) or after Ramadan (IRR: 1.000; 95% CI: 0.992–1.007). No significant change in antibiotic prescription patterns was identified in patients with diabetes during Ramadan (IRR: 0.996; 95% CI: 0.988–1.005) or after Ramadan (IRR:1.003, 95% CI: 0.994–1.012).

## Discussion

To our knowledge, this is the first study carried out to determine patterns of antibiotic prescriptions before, during, and after Ramadan for a predominantly Muslim community in the UK. In this large population-based study, we have demonstrated that there was an increase in antibiotic prescribing following Ramadan (compared to during Ramadan) among people of Pakistani or Bangladeshi origin within the UK; however, this did not remain significant when corrected for antibiotic prescriptions in the white population. Importantly, the Ramadan period was not associated with altered prescription patterns among older adults (>60) or among those with diabetes, the two groups with the greatest potential infection risk.

The effects that fasting during Ramadan exerts on the immune system have been previously studied using indices of basal inflammation and its potential to respond to infection. However, these studies have typically observed only small samples of healthy individuals, yielding conflicting results; this may be partially explained by the timing and methods used for assessment [3, 4]. The significance of transient changes in basal inflammation is not clear, and the response to infection during Ramadan fasting has not been widely studied. However, in vitro assays from small numbers of people have demonstrated a continued, and possibly increased, ability to respond to infection during Ramadan fasting [30, 31].

Our findings suggest an increase in infection rates after Ramadan. However, it is unknown whether this result is from changes in immune regulation, possible increased exposure to infectious agents, a combination of both, or behaviour modification. Meals during Ramadan tend to be rich in sugar and fat, which can be associated with an increased risk of infection [32]. Practising Muslims participate in many social events during and after Ramadan, including the celebration of Eid, marking the end of fasting. It is possible that these broader social interactions, which increase potential exposure to infection, may also contribute to our findings. Further research is required to determine which of these elements of Ramadan contribute to elevated risk. However, since increased risk of infection after Ramadan was not seen when corrected for the white population, there may be additional confounders, such as seasonal variation, that contribute to this potential.

The important finding that older adults (over 60 years old) and people with diabetes were not affected by the Ramadan period should be interpreted with caution. Behavioural modifications, such as reducing the number of fasting days or not observing the fast due to pre-existing conditions, may explain the absence of an effect. Indications that diabetics who have additional comorbidities are avoiding fasting is supported by hospital data [33]. However, other studies report that the majority of diabetic Muslims are participating in Ramadan fasting [16, 34]. The effects of diabetes [35] and age [36] on the immune system are well-established, including increased susceptibility to infection, especially severe infection. Nevertheless, any potential modulation of this effect by Ramadan fasting is unknown. Extrapolating data from non-Ramadan intermittent fasting (as undertaken by diabetics to achieve weight loss) is difficult, given the potential for significant confounders such as exercise modulation, caloric reduction, differences in hydration status, and other health-promoting behaviours that may be part of a weight-loss strategy. Further investigations are warranted on the effects of Ramadan fasting on the older and diabetic Muslim population.

While we did not demonstrate a significant difference in antibiotic prescribing during or after Ramadan among those who observe and those who do not observe, we considered the possibility that prescriptions of antibiotics during Ramadan may decrease due to fewer primary care visits for mild illnesses during that period. However, this is not supported by a large US-based study on primary care visits [37] that actually showed an increase during the Ramadan period.

Our epidemiological approach to the question of whether Ramadan fasting is associated with an increased risk of bacterial infection has advantages over previously published work, in that we focus on clinically significant bacterial infections requiring antibiotic therapy. Further strengths of our study include the extensive study period, large sample size, and the use of a control group that does not typically observe Ramadan. Another key strength of this study is our use of interrupted time series analysis on routinely collected data, which allowed for tracking changes during the different periods.

Some limitations existed in our study. One of the limitations was that our analyses did not account for seasonal change. Ramadan moves 11 days forward every year, and it can take up to 33 years to occur in all seasons. Our study used ten years of data, during which Ramadan occurred in summer and autumn (June to mid-October). Including data from years where Ramadan took place in other months could have aided our understanding of whether temperature, hours of daylight, and hours of sleep play a role in the risk of infection. Another limitation was that we could not confirm who in our exposed group definitely took part in Ramadan fasting, leading to ecological fallacy. Our assumption of fasting within the Pakistani and Bangladeshi populations compared to the broader UK population was based on estimates that more than 90% of people from these ethnic backgrounds are Muslims [38]. According to a Pew Research Centre survey of more than 38,000 Muslims around the world, most Muslims practise fasting during Ramadan [10]. Therefore, we assume that our exposed group accurately represented practising Muslims. Given that we only investigated the Pakistani and Bangladeshi population in the UK, the results from our study may not reflect other Muslim communities within the country. Moreover, the generalisability of the results is limited due to variations in the prevalence of pre-existing conditions, such as diabetes, and of infectious agents circulating in Muslim populations in other countries. Generalisability is also affected by differing fasting rituals, cultural traditions, daylight hours, and temperatures in other countries during Ramadan.

The absence of a clear effect is encouraging in terms of the safety of fasting, although at-risk groups should note that additional risks may remain, including complications from diabetes and cardiovascular disease, which were beyond the scope of this study. Currently, there is no evidence that Ramadan fasting itself increases the risk of contracting bacterial infections within the studied population, although the generalisability to other populations (including in warmer climates) is uncertain. Further research on this topic in other communities and countries is necessary, in order to account for factors such as Ramadan season, age, gender, and comorbidity.

## Supporting information

S1 Appendix Assumptions. (DOCX)

S2 Appendix Dataset structure. (XLSX)

S3 Appendix Parameter estimates. (DOCX)
