# Supplementary material for: Ramadan is not associated with increased infection risk in Pakistani and Bangladeshi populations: Findings from controlled interrupted time series analysis of UK primary care data
Source: PLoS One. 2022 Jan 13;17(1):e0262530. doi: 10.1371/journal.pone.0262530 (PMC8757987; doi:10.1371/journal.pone.0262530)
Supplement: S1 Appendix — (DOCX) [file pone.0262530.s001.docx]

Assumptions:

Interrupted time series design’s validity rests on the following assumptions[1]:

- **Assumption 1:** The expectation of the pre intervention level and trend would be the same irrespective of whether the sample received the treatment

We checked this assumption through applying single interrupted time series to the targeted group (Pakistani/Bangladeshi) and compared it to the control group (white) that were not exposed to Ramadan.

The slops in both models were similar. However, levels were different. This is expected as the size of the two populations is different.

**Before vs during Ramadan**

| Model parameters | Pakistani/Bangladeshi | White |
| --- | --- | --- |
| level | 513.45 (95%CI:481.73-547.27) | 32799.25 (95%CI:31116.01-34573.54) |
| Slop | 1.00 (95%CI:0.99-1.00) | 0.99 (95%CI:0.99-1.00) |

**During vs after Ramadan**

| Model parameters | Pakistani/Bangladeshi | White |
| --- | --- | --- |
| level | 502.72 (95%CI:466.45-541.82) | 32119.015(95%CI:30451.88-33877.40) |
| Slop | 0.99 (95%CI:0.99-0.99) | 1.000 (95%CI:0.99-1.0) |

**Assumption 2:** In the absence of the intervention, the post intervention trendline would have been equivalent in expectation to an extrapolated pre intervention trend.

We checked this assumption through comparing Intercepts and slops in the different time series (before, during and after Ramadan) in the control group (white) that were not exposed to Ramadan. Both intercepts and slops are similar in all period.

There are 3 different types of models we have fitted:

Model A: **Before Ramadan series (30 days)**

Model B: **During Ramadan series (30 days)**

Model C: **After Ramadan (30 days)**

| Model parameters | **Model A** | **Model B** | **Model C** |
| --- | --- | --- | --- |
| level | 32799.25 (95%CI: 31223.73-34454.27) | 32119.01 (95%CI: 30363.24-33976.31) | 33008.978 (95%CI: 31417.44- 34681.14) |
| slop | 0.999 (95%CI: 0.996 - 1.002) | 1.0 (95%CI: 0.99- 1.004) | 1.001 (95%CI: 0.99-1.004) |

**Sensitivity analysis**

We conducted a sensitivity analysis with incorporating for seasonality (daily temperature) for selected years and found that temperature did not affect the results significantly.

**Before vs during Ramadan**

1. **the model results without incorporating for seasonality**

| Year | Model parameters | Pakistani/Bangladeshi | White |
| --- | --- | --- | --- |
| 2007 | level | 0.96 (95%CI:0.48-1.93) | 0.90 (95%CI:0.45-1.79) |
|  | Slop | 0.997(95%CI:0.95-1.03) | 1.01 (95%CI:0.97-1.05) |
| 2008 | level | 1.37 (95%CI:0.65-2.9) | 1.35 (95%CI:0.66-2.78) |
|  | Slop | 1.00 (95%CI:0.96-1.04) | 1.00 (95%CI:0.96-1.04) |
| 2009 | level | 0.75 (95%CI:0.37-1.53) | 0.79 (95%CI:0.39-1.61) |
|  | Slop | 0.98 (95%CI:0.94-1.03) | 0.99 (95%CI:0.95-1.03) |
| 2010 | level | 0.91 (95%CI:0.45-1.82) | 1.01 (95%CI:0.52-1.98) |
|  | Slop | 1.01 (95%CI:0.97-1.05) | 1.01 (95%CI:0.97-1.05) |
| 2011 | level | 1.42 (95%CI:0.69-2.90) | 1.32 (95%CI:0.66-2.65) |
|  | Slop | 0.98 (95%CI:0.94-1.03) | 0.98 (95%CI:0.94-1.03) |

1. **the model results with incorporating for seasonality (daily temperature)**

| Year | Model parameters | Pakistani/Bangladeshi | White |
| --- | --- | --- | --- |
| 2007 | level | 1.29 (95%CI:0.58-2.87) | 1.21 (95%CI:0.56-2.64) |
|  | Slop | 1.00 (95%CI:0.96-1.04) | 1.02 (95%CI:0.97-1.05) |
| 2008 | level | 1.30 (95%CI:0.59-2.83) | 1.32 (95%CI:0.62-2.80) |
|  | Slop | 1.00 (95%CI:0.96-1.04) | 1.00 (95%CI:0.96-1.04) |
| 2009 | level | 0.74 (95%CI:0.35-1.55) | 0.80 (95%CI:0.38-1.67) |
|  | Slop | 0.98 (95%CI:0.94-1.03) | 0.99 (95%CI:0.95-1.04) |
| 2010 | level | 0.88 (95%CI:0.43-1.76) | 0.96 (95%CI:0.49-1.87) |
|  | Slop | 1.01 (95%CI0.97-1.05) | 1.00 (95%CI:0.96-1.04) |
| 2011 | level | 1.55 (95%CI:0.73-3.29) | 1.45 (95%CI:0.70-3.0) |
|  | Slop | 0.98 (95%CI:0.94-1.03) | 0.98 (95%CI:0.95-1.03) |

**During vs after Ramadan**

1. **the model results without incorporating for seasonality**

| Year | Model parameters | Pakistani/Bangladeshi | White |
| --- | --- | --- | --- |
| 2007 | level | 1.00 (95%CI:0.48- 2.11) | 0.81 (95%CI:0.41-1.61) |
|  | Slop | 1.00 (95%CI:0.96-1.04) | 0.98 (95%CI:0.94-1.02) |
| 2008 | level | 1.17 (95%CI:0.60-2.29) | 1.08 (95%CI:0.56-2.06) |
|  | Slop | 0.99 (95%CI:0.96-1.03) | 1.00 (95%CI:0.96-1.04) |
| 2009 | level | 1.77 (95%CI:0.86-3.64) | 1.28 (95%CI:0.63-2.56) |
|  | Slop | 0.99 (95%CI:0.95-1.03) | 0.99 (95%CI:0.95-1.03) |
| 2010 | level | 0.94 (95%CI:0.47-1.88) | 0.92 (95%CI:0.46-1.81) |
|  | Slop | 0.99 (95%CI:0.96-1.04) | 1.00 (95%CI:0.96-1.04) |
| 2011 | level | 1.48 (95%:CI:0.72-3.00) | 1.218 (95%CI:0.62-2.39) |
|  | Slop | 1.02 (95%CI:0.98-1.06) | 1.017 (95%CI:0.98-1.05) |

1. **the model results with incorporating for seasonality (daily temperature)**

| Year | Model parameters | Pakistani/Bangladeshi | White |
| --- | --- | --- | --- |
| 2007 | level | 1.03 (95%CI:0.49-2.16) | 0.82 (95%CI:0.41-1.65) |
|  | Slop | 1.01 (95%CI:0.96-1.05) | 0.99 (95%CI:0.95-1.03) |
| 2008 | level | 1.08 (95%CI:0.54-2.17) | 1.02 (95%CI:0.52-2.01) |
|  | Slop | 0.99 (95%CI:0.95-1.03) | 1.00 (95%CI:0.96-1.03) |
| 2009 | level | 1.72 (95%CI:0.82-3.61) | 1.24 (95%CI:0.60-2.55) |
|  | Slop | 0.98 (95%CI:0.94-1.03) | 0.99 (95%CI:0.94-1.03) |
| 2010 | level | 0.92 (95%CI:0.46-1.85) | 0.90 (95%CI:0.45-1.79) |
|  | Slop | 1.00 (95%CI:0.96-1.04) | 1.00 (95%CI:0.96-1.04) |
| 2011 | level | 1.58 (95%CI:0.76-3.31) | 1.01 (95%CI:0.98-1.05) |
|  | Slop | 1.02 (95%CI:0.98-1.06) | 0.97 (95%CI:0.93-1.03) |

1. Baicker K, Svoronos T. Testing the Validity of the Single Interrupted Time Series Design. ERN: Hypothesis Testing (Topic). 2019.
