# Supplementary material for: Ramadan is not associated with increased infection risk in Pakistani and Bangladeshi populations: Findings from controlled interrupted time series analysis of UK primary care data
Source: PLoS One. 2022 Jan 13;17(1):e0262530. doi: 10.1371/journal.pone.0262530 (PMC8757987; doi:10.1371/journal.pone.0262530)
Supplement: S3 Appendix — (DOCX) [file pone.0262530.s003.docx]

**Parameter estimates**

1. Single interrupted time series (Pakistani/Bangladeshi)
   1. Before vs during Ramadan

| Parameter | Interpretation | Estimation (95%CI) |
| --- | --- | --- |
| *β_0_* | Intercept | 513.459 (95%CI: 481.731-547.276) |
| *β_1_* | Pre-trend | 1.000 (95%CI:0.996-1.003 |
| *β_2_* | Post-level change | 0.990 (95%CI: 0.905-1.083) |
| *β_3_* | Post-trend change | 0.995 (95%CI:0.990-1.001) |

- 1. During vs during Ramadan

| Parameter | Interpretation | Estimation (95%CI) |
| --- | --- | --- |
| *β_0_* | Intercept | 502.725 (95%CI:466.451-541.820) |
| *β_1_* | Pre-trend | 0.995 (95%CI:0.991-0.999) |
| *β_2_* | Post-level change | 1.165 (95%CI:1.050-1.292) |
| *β_3_* | Post-trend change | 1.007 (95%CI:1.001-1.013) |

1. Single interrupted time series (white)
   1. Before vs during Ramadan

| Parameter | Interpretation | Estimation (95%CI) |
| --- | --- | --- |
| *β_0_* | Intercept | 32799.253 (95%CI:31116.019-34573.543) |
| *β_1_* | Pre-trend | 0.999 (95%CI:0.996-1.002) |
| *β_2_* | Post-level change | 1.005 (95%CI:0.934-1.081) |
| *β_3_* | Post-trend change | 1.001 (95%CI:0.997-1.006) |

- 1. During vs during Ramadan

| Parameter | Interpretation | Estimation (95%CI) |
| --- | --- | --- |
| *β_0_* | Intercept | 32119.015 (95%CI:30451.889-33877.409) |
| *β_1_* | Pre-trend | 1.000 (95%CI:0.997-1.003) |
| *β_2_* | Post-level change | 1.014 (95%CI:0.943-1.091) |
| *β_3_* | Post-trend change | 1.001 (95%CI:0.996-1.005) |

1. Controlled interrupted time series
   1. Before vs during Ramadan

| Parameter | Interpretation | Estimation (95%CI) |
| --- | --- | --- |
| *β_0_* | Intercept | 32801.625 (95%CI:31086.900-34610.933) |
| *β_1_* | Control pre-trend | 0.999 (95%CI: 0.996-1.002) |
| *β_2_* | Control post level change | 1.005 (95%CI: 0.933- 1.083) |
| *β_3_* | Post-trend change | 1.001 (95%CI: 0.997- 1.006) |
| *β_4_* | Treatment/control pre-trend difference | 0.016 (95%CI: 0.014-0.017) |
| *β_5_* | Treatment/control pre-level difference | 1.001 (95%CI: 0.996 -1.005) |
| *β_6_* | Treatment/control post level difference | 0.985 (95%CI: 0.879-1.104) |
| *β_7_* | Treatment/control change in slope | 0.994 (95%CI: 0.988-1.001) |

- 1. During vs during Ramadan

| Parameter | Interpretation | Estimation (95%CI) |
| --- | --- | --- |
| *β_0_* | Intercept | 32120.057 (95%CI: 30321.572-34025.218) |
| *β_1_* | Control pre-trend | 1.000 (95%CI: 0.997-1.004) |
| *β_2_* | Control post level change | 1.015 (95%CI: 0.937-1.098) |
| *β_3_* | Post-trend change | 1.001 (95%CI: 0.996-1.005) |
| *β_4_* | Treatment/control pre-trend difference | 0.016 (95%CI: 0.014-0.017) |
| *β_5_* | Treatment/control pre-level difference | 0.995 (95%CI: 0.990-1.000 ) |
| *β_6_* | Treatment/control post level difference | 1.148 (95%CI: 1.017-1.297) |
| *β_7_* | Treatment/control change in slope | 1.006 (95%CI: 0.999-1.013) |
